# Shape‐Morphing Photoresponsive Hydrogels Reveal Dynamic Topographical Conditioning of Fibroblasts

**DOI:** 10.1002/advs.202303136

**Published:** 2023-09-23

**Authors:** Maaike Bril, Aref Saberi, Ignasi Jorba, Mark C. van Turnhout, Cecilia M. Sahlgren, Carlijn V.C. Bouten, Albert P.H.J. Schenning, Nicholas A. Kurniawan

**Affiliations:** ^1^ Department of Biomedical Engineering Eindhoven University of Technology Eindhoven 5600 MB The Netherlands; ^2^ Institute for Complex Molecular Systems Eindhoven University of Technology Eindhoven 5600 MB The Netherlands; ^3^ Faculty of Science and Engineering Åbo Akademi University Turku FI‐20520 Finland; ^4^ Department of Chemical Engineering & Chemistry Eindhoven University of Technology Eindhoven 5612 AE The Netherlands

**Keywords:** dynamic topographies, light‐responsive hydrogel, mechanotransduction, nucleus

## Abstract

The extracellular environment defines a physical boundary condition with which cells interact. However, to date, cell response to geometrical environmental cues is largely studied in static settings, which fails to capture the spatiotemporally varying cues cells receive in native tissues. Here, a photoresponsive spiropyran‐based hydrogel is presented as a dynamic, cell‐compatible, and reconfigurable substrate. Local stimulation with blue light (455 nm) alters hydrogel swelling, resulting in on‐demand reversible micrometer‐scale changes in surface topography within 15 min, allowing investigation into cell response to controlled geometry actuations. At short term (1 h after actuation), fibroblasts respond to multiple rounds of recurring topographical changes by reorganizing their nucleus and focal adhesions (FA). FAs form primarily at the dynamic regions of the hydrogel; however, this propensity is abolished when the topography is reconfigured from grooves to pits, demonstrating that topographical changes dynamically condition fibroblasts. Further, this dynamic conditioning is found to be associated with long‐term (72 h) maintenance of focal adhesions and epigenetic modifications. Overall, this study offers a new approach to dissect the dynamic interplay between cells and their microenvironment and shines a new light on the cell's ability to adapt to topographical changes through FA‐based mechanotransduction.

## Graphical abstract

This study presents a cell‐compatible hydrogel‐based platform that allows reconfiguration of the topography of the cellular environment on demand using illumination. It demonstrates that fibroblasts sense the dynamic topographies and suggest that cells are capable of “remembering” previous topographies via the formation of focal adhesion complexes on the dynamic regions of the substrate, possibly to prepare for future biophysical events.

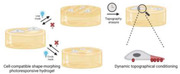

## 1 Introduction

Adherent cells are surrounded by an extracellular matrix (ECM), which presents physical cues that influence cell behavior, such as cell migration,^[^ ^1^ ^]^ differentiation,^[^ ^2^, ^3^ ^]^ and proliferation,^[^ ^4^ ^]^ ultimately contributing to tissue morphogenesis and homeostasis.^[^ ^5^, ^6^ ^]^ The physiological functions of tissues, such as the blood pumping of the heart, the deformability of the skin, and the load‐bearing capacity of the tendon, often necessitate specific geometrical and topographical configuration of the ECM, for example in the form of microscale fibers and mesoscale curvature (scale of hundreds of µm).^[^ ^7^, ^8^, ^9^, ^10^, ^11^ ^]^ Cells can sense the mechanical and topographical properties of their microenvironment by forming multiprotein adherence complexes at the cell membrane, called focal adhesions (FAs).^[^ ^12^ ^]^ On the cellular side, these focal adhesions are in direct contact with the actin cytoskeleton, allowing mechanical events to be transduced intracellularly into biochemical signals (termed mechanotransduction). In turn, the cytoskeleton is connected to the nuclear envelope, allowing forces to reach and alter the nucleus.^[^ ^13^ ^]^ Recent studies showed that physical cues, such as stiffness,^[^ ^14^ ^]^ curvature,^[^ ^15^ ^]^ and topography,^[^ ^16^, ^17^ ^]^ can affect chromatin condensation and induce epigenetic modifications, thereby influencing gene expression and maintaining genome stability.^[^ ^18^, ^19^, ^20^ ^]^

The interaction between cells and the ECM is highly dynamic and reciprocal. On the one hand, environmental stimuli are presented to cells in a spatiotemporal manner to, for example, guide embryogenesis and tissue homeostasis.^[^ ^5^, ^6^, ^21^, ^22^ ^]^ On the other hand, cells themselves can remodel their matrix, for example during wound healing and cell migration.^[^ ^6^, ^23^, ^24^, ^25^ ^]^ This remodeling process results in continuous changes in tissue organization, architecture, and geometry, which in turn are crucial for tissue function.^[^ ^22^, ^26^, ^27^, ^28^ ^]^ In fact, changes in tissue architecture and loss of matrix organization are widely associated with pathologies,^[^ ^27^ ^]^ yet the causal relationship between divergent cell behavior and tissue dynamics remains poorly understood.

Although key cellular mechanotransduction components involved in cellular sensing of physical and geometrical cues have been discovered,^[^ ^29^, ^30^, ^31^ ^]^ the dynamic nature of these cues has been largely overlooked, primarily due to the lack of an experimental platform that allows systematic interrogation of cell sensing of dynamic topographical changes. Additionally, these dynamic cell‐ECM interactions take place over a variety of time scales. For example, ECM‐ligand binding by the integrin receptor happens in seconds, remodeling of the cytoskeleton and FAs requires minutes to hours, whereas meso‐ and macroscale remodeling of the ECM and tissue organization takes days till weeks.^[^ ^32^, ^33^, ^34^, ^35^ ^]^ Studying these dynamic events while being able to monitor the underlying cellular processes therefore requires new tools that allow spatiotemporal control of the dynamic changes in the cellular environment.

Recent advances in materials engineering has enabled the development of dynamic cell culture platforms whose properties can be modified spatiotemporally using external stimuli.^[^ ^6^ ^]^ Surface topography can be altered by using temperature‐sensitive polycaprolactone shape‐memory polymers,^[^ ^36^ ^]^ strain‐responsive polydimethylsiloxane (PDMS),^[^ ^37^, ^38^ ^]^ or by using photoresponsive azobenzene‐based systems.^[^ ^39^, ^40^, ^41^, ^42^ ^]^ Photoresponsive platforms are attractive since light provides a local and contact‐free remote‐control possibility with high spatial resolution and tunability. In the last decade, azobenzene‐based materials, such as liquid crystal polymer networks and azopolymer films, have been reported to induce topographical changes.^[^ ^39^, ^40^, ^41^, ^42^, ^43^, ^44^ ^]^ In the presence of light, the azobenzene group undergoes a *trans*‐to‐*cis* conformational change, resulting in mass migration of the polymer network and the subsequent formation of protrusions. The induced surface topographies were shown to instruct and guide cell migration and alignment,^[^ ^43^ ^]^ although this was also accompanied with a significant change in surface roughness^[^ ^42^ ^]^ and generation of unwanted surface features upon illumination in a liquid environment.^[^ ^39^, ^40^ ^]^ Although liquid crystal networks do not suffer from these side effects, it is challenging to change their topographies in situ with desired height changes.^[^ ^45^ ^]^ In short, despite their potential, the use of these materials as dynamic cell culture substrates has been limited due to unwanted side effects upon material actuation, challenging production routes, or the requirement of specialized equipment.

Here, we present a facile approach to develop a light‐responsive cell culture platform based on a spiropyran‐containing poly(N‐isopropylacrylamide) (Sp‐pNIPAM) hydrogel.^[^ ^46^ ^]^ We demonstrate that this set‐up allows one to expose living cells on demand to a variety of reversible dynamic topographies that can be isotropic as well as anisotropic, with user‐defined timespan, and without changing any other material property. Here, we refer to “dynamic” as an induced change in material geometry (within a time scale of minutes), whereas “static” conditions contain no change in material properties. To show its suitability for biological applications, we seeded dermal fibroblasts, which in living tissues are exposed to continuous topographical changes due to bodily movements, stretching of the skin, wound healing, and skin aging.^[^ ^35^, ^47^, ^48^ ^]^ By exposing dermal fibroblasts to multiple rounds of changing topographies, we found that the cells adapted their orientation and morphology, FA formation, and nuclear material in a location‐dependent manner, providing first evidence of a dynamic topographical responsiveness of cells. This easy‐to‐use light‐responsive cell culture platform thus provides a new and versatile way to shine light on the dynamic interplay between matrix topography and cells, the effect of multiple rounds of changing topographies and geometries, and the role and timing of the underlying mechanotransductive pathways.

## 2 Results

### 2.1 SBS‐Sp‐pNIPAM Hydrogels as Reversible Light‐Responsive Substrates to Create Dynamic Topographies

**Figure 1:**
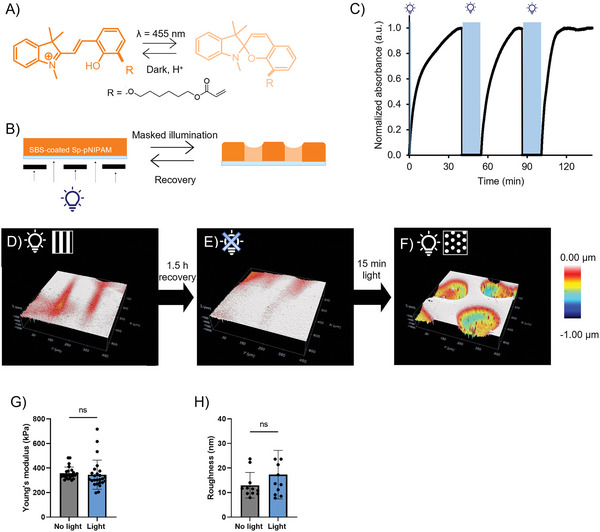
Schematic representation and characterization of the photoresponsive cell culture platform. A) Photoisomerization of the hydrophilic protonated merocyanine to the hydrophobic spiropyran (Sp). B) Masked illumination (λ = 455 nm) of the SBS‐coated spiropyran‐containing pNIPAM hydrogel results in the formation of a topographical pattern. C) UV–vis spectroscopy showed the reversible character of the substrate under cell culture conditions. The change in absorption at 402 nm was measured during three successive 15 min exposure steps at 455 nm. D) Masked illumination (455 nm, 15 min) induced grooves, as observed by optical profilometry. E) Recovery of the substrate in the dark resulted in a flat surface. F) Another round of masked illumination with the same sample (455 nm, 15 min) resulted in a new type of surface topography. G) Substrate stiffness, before and after prolonged blue light exposure (6 h), as measured with nanoindentation. Paired *t*‐test, ns = not significant. H) Substrate roughness, measured with AFM in contact mode, before and after illumination (455 nm, 15 min). Paired *t*‐test, ns = not significant.

To assemble a photoresponsive cell culture platform, we produced surface‐constrained 120‐µm‐thick Sp‐pNIPAM hydrogels (E = 56.44 ± 1.12 kPa) (**Figure** **1**; Figure S1, Supporting Information). Upon blue light illumination (455 nm), the stable, hydrophilic protonated merocyanine form isomerizes to the hydrophobic spiropyran form (Figure 1A), changing the hydration of the polymer network and resulting in hydrogel shrinkage (Figure 1B). Since the photoisomerization requires an acidic environment, a thin but dense elastomeric styrene‐butadiene‐styrene (SBS, 100 mg mL^−1^) layer (thickness of 5.75 ± 2.00 µm, see Figure S2, Supporting Information) was spin‐coated on top of the hydrogel to provide chemical resistance against the pH buffers present in cell culture medium. Upon illumination through a mask, the water in the exposed areas of the SBS‐Sp‐pNIPAM hydrogel migrates to the unexposed areas, resulting in local and temporal hydrogel shrinkage. The photoresponsiveness of these constructs was first confirmed using UV–vis spectroscopy. Absorption measurements showed an absorption maximum at λ = 402 nm, which corresponds to the presence of the protonated merocyanine (Figure S3, Supporting Information). When the sample was illuminated with blue light (455 nm, 15 min) in culture medium, the protonated merocyanine absorbance at 402 nm decreased, indicating that the protonated merocyanine was isomerized to the spiropyran form. Within 1 h, the absorption peak returned to the original value, demonstrating the recovery of the protonated merocyanine. Three successive illumination rounds showed that the recovery is reversible and repeatable (Figure 1C).

To introduce surface topographies with desired features, hydrogel constructs were illuminated (455 nm, 15 min) through a mask with patterns of open lines or circles (Figure 1B). Optical interferometry measurements showed that a variety of topographies, which can be isotropic or anisotropic, can be successively generated in the same sample (Figure 1D–F). To illustrate this feature, we first generated constructs with a rectangular topographic pattern (90 µm wide grooves, 350 nm high) (Figure 1D), after which the construct was allowed to recover from material deformation in the dark for 1.5 h. Profilometry measurements confirmed the recovery of the flat surface (Figure 1E), after which a new type of topography can be introduced. Within the same sample, we introduced 150 µm wide concave pits with a height of ≈920 nm (Figure 1F), showing the versatility and flexibility of this masked exposure technique. The height of the introduced topography can be easily and orthogonally tuned by varying the amount of the crosslinker MBIS in the SBS‐Sp‐pNIPAM hydrogel. For example, lowering the MBIS concentration to 0.5% resulted in structures with a height of 4 µm (Figure S4, Supporting Information). It should be noted that inducing topographies too close to each other (< 50 µm) may result in overlap of the shrunken areas (Figure S5, Supporting Information).

Since cells not only sense topography, but also respond to other mechanical cues such as strain, material stiffness, and roughness, we verified whether these parameters remained unchanged after illumination. To investigate whether the induction of topography lead to lateral strain across the hydrogel surface, surface contour length before and after introduction of the topography was measured. The measured distances before and after actuation showed negligible surface strain (<1% strain on average; Figure S6 and Table S1, Supporting Information). Nanoindentation measurements further confirmed that prolonged illumination (6 h) did not significantly change the Young's modulus of the SBS‐Sp‐pNIPAM hydrogels (E = 349 ± 63 kPa; Figure 1G; Figure S7, Supporting Information). Additionally, atomic force microscopy (AFM) measurements revealed that surface roughness of the substrate was not altered upon illumination (Rq = 15 ± 5 nm; Figure 1H; Figure S8, Supporting Information). Together, these data indicate that the presented system successfully overcomes the technical challenge of achieving desired dynamic changes in material topography without unwanted material side effects.

### 2.2 Fibroblasts Adapt their Morphology upon Dynamic Topographical Actuation

To investigate how dynamic topographies affect cell behavior, we assessed normal human dermal fibroblast (nhDF) morphology after one and two rounds of topographical changes. Photoisomerization of SBS‐Sp‐pNIPAM was confirmed after UV‐sterilization, and UV‐light seems to enhance the recovery process, which is not considered to be problematic (Figure S9, Supporting Information). To allow cell adhesion, the hydrogel was coated with fibronectin. Fluorescence signal from rhodamine‐labeled fibronectin indicated a homogenous coating of the hydrogel surface (Figure S10, Supporting Information), and fibroblasts were able to adhere normally on the fibronectin‐coated SBS‐Sp‐pNIPAM hydrogel. No significant difference in cell viability was observed before and after (masked) illumination, and a viability of ≈90% was maintained throughout the experiment (Figure S11, Supporting Information). To check whether blue light illumination may have induced DNA damage, we immunolabeled gamma‐H2AX, which forms when histone H2AX is phosphorylated upon the formation of DNA double‐stranded breaks (Supporting Information).^[^ ^18^, ^49^ ^]^ No gamma‐H2AX foci were observed, confirming the absence of DNA damage upon our illumination protocol (Figure S12, Supporting Information).

**Figure 2:**
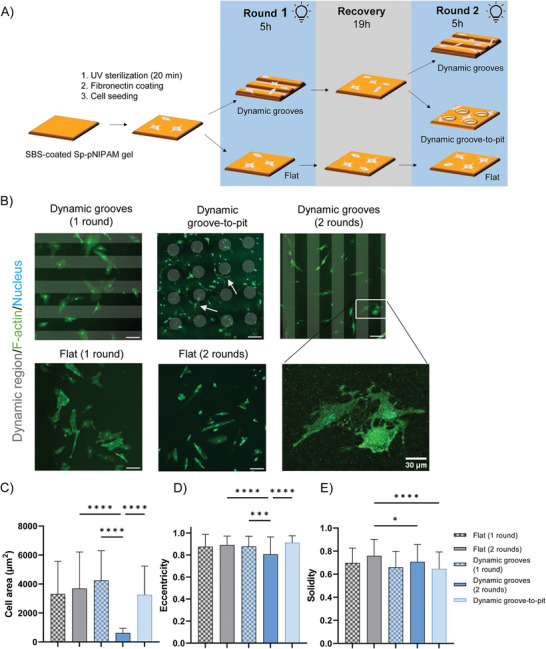
Subjecting cells to dynamic topographies. A) Schematic overview of SBS‐Sp‐pNIPAM sample preparation and patterning protocol for cell culture applications. B) Immunofluorescence image of nhDFs cultured on dynamic topographies. Shaded areas: regions of the hydrogel with induced dynamic topography, green: F‐actin (phalloidin), blue: nuclei (DAPI). Scale bar is 100 µm. Arrows: cells wrap and align with the concave pits. Marked region shows a zoom‐in of the F‐actin cytoskeleton. Scalebar = 30 µm. C) Cell area. D) Cell eccentricity. E) Cell solidity. Mean with SD, n ≥ 31 analyzed cells. One‐way ANOVA (^*^ *p* ≤ 0.05, ^**^ *p* ≤ 0.01, ^***^ *p* ≤ 0.001, ^****^ *p* ≤ 0.0001).

We then subjected fibroblasts to dynamic topographical changes and analyzed the resulting cellular organization and morphology. First, the cells were allowed to adhere to the substrate, after which they were exposed to light‐induced anisotropic patterns (90 µm wide grooves) for 5 h. Subsequently, the cells were either fixed, or, after overnight recovery, subjected to a second round of flat‐to‐anisotropic grooves (**Figure** **2**A). Due to the fluorescence of the protonated merocyanine, patterns could be visualized using confocal fluorescence microscopy, which also confirmed generation of surface topographies in a standard cell‐culture atmosphere (humidified 37°C) (Figure S13, Supporting Information). Upon dynamic topographical changes, cells remained adhered to the hydrogel surface, but were found to alter their morphologies (Figure 2B and Movie S1, Supporting Information). After one round of actuation, cell area slightly increased in comparison to cell area on the flat control (Figure 2C), without altering cell elongation (parameterized as eccentricity, Figure 2D).

To date, the cell response to multiple topographical changes has not been investigated. Interestingly, we found that a second round of substrate actuation resulted in significantly smaller cell area (Figure 2C) and reduced cell elongation (Figure 2D), while showing an increased tendency to align either parallel or perpendicular to the introduced anisotropic topographical pattern (Figure S14, Supporting Information). Additionally, cells displayed no clear stress fibers (Figure 2B, zoom‐in), which are normally present in these cells. Thus, we hypothesized that the dynamics of the actin cytoskeleton is critical in responding to the recurring dynamic topographies, and that stabilization of the actin cytoskeleton could help the cells in responding to and withstanding the dynamic topographical events. Indeed, promotion of actin filament polymerization with Jasplakinolide (added 15 min before material actuation) recovered cell area on the dynamic SBS‐Sp‐pNIPAM substrates back to the level on flat controls and resulted in more elongated cells (Figure S15, Supporting Information), indicating the active role of the actin cytoskeleton in responding to dynamic topographies.

While the above experiments shed light on the cells’ response to recurring changes of the same type of anisotropic topography (grooves), our approach also allows us to create different types of material topographies within the same sample. This will enable us to study the effect of in situ changing topographies, which is relevant in vivo, for example during the loss of anisotropic matrix organization in pathological events such as myocardial infarction,^[^ ^50^ ^]^ tendinopathies,^[^ ^51^ ^]^ and tumorigenesis.^[^ ^27^, ^52^ ^]^ To demonstrate this possibility, we reconfigured the substrate topography from anisotropic grooves to isotropic features (concave pits) by switching the mask between the two rounds of actuation (Figure 2A). The result showed that fibroblasts adapted their morphology by increasing cell area (Figure 2C) and becoming more elongated (Figure 2D) compared to cells cultured on the dynamic groove hydrogels. Additionally, we note that the cells appeared to avoid the dynamic concave pits (Figure 2B). This is in contrast to what has been previously observed on substrates with static concave pits (30 µm wide), where cells were more likely to be found on the static concave regions.^[^ ^53^ ^]^ The findings using our shape‐morphing topographies demonstrate that cells can actively sense and respond to reconfigured dynamic topographical changes.

### 2.3 Focal Adhesion Reorganization in Response to Dynamic Topographies

**Figure 3:**
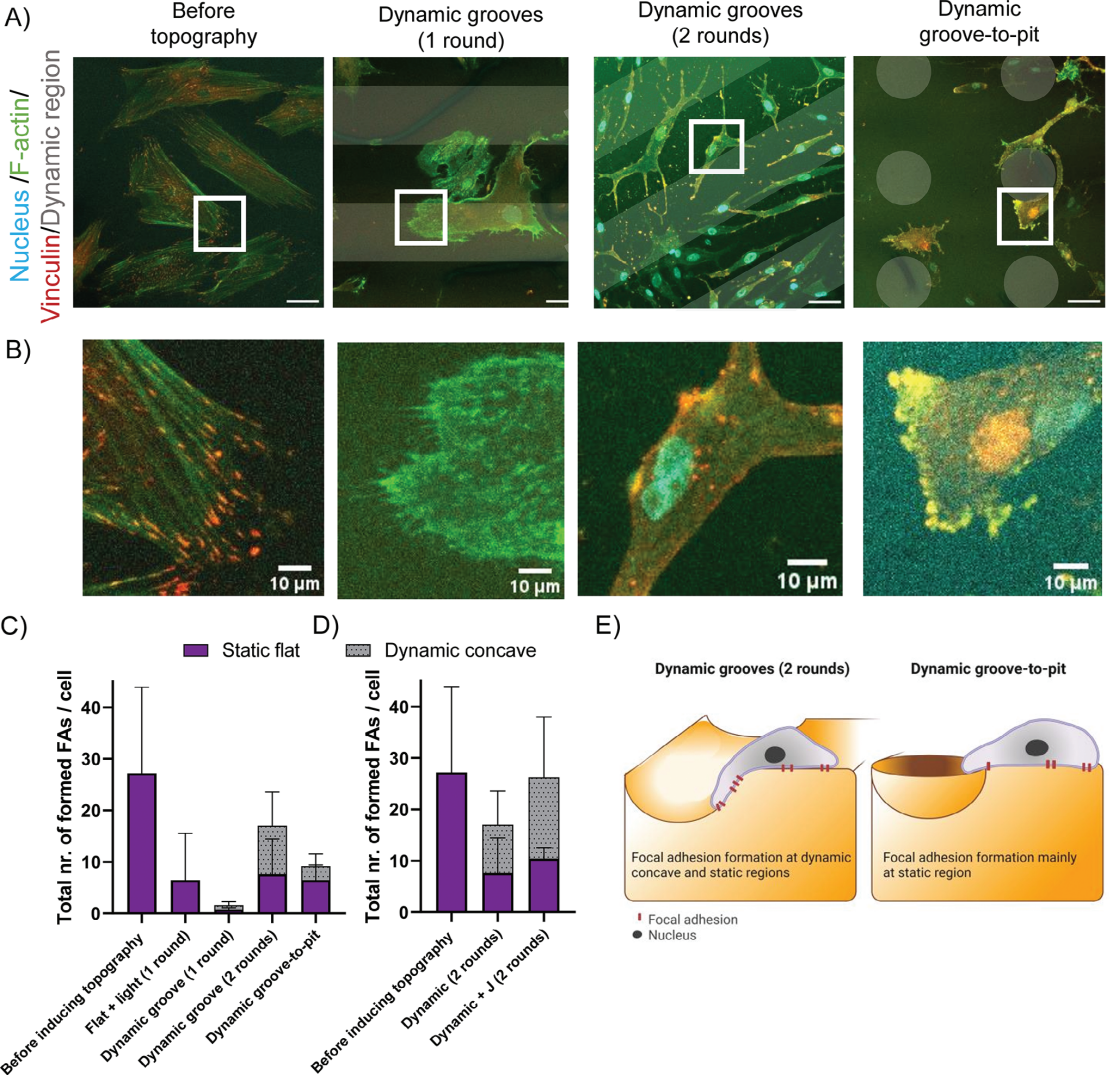
Focal adhesion formation on dynamic topographies. A) Representative confocal images of nhDFs before and after inducing dynamic topographies. After subjecting cells to the indicted topography, cells were fixed and stained for F‐actin (green), vinculin (red), and nuclei (blue). The dynamic regions of the gel are shown as shaded areas. Scale bar is 50 µm. B) Zoom‐in images of the indicated regions from panel A. Scale bar is 10 µm. C) Quantification of focal adhesion formation per cell per region of the hydrogel (dynamic or static). Analyzed cells before inducing topography: 37 cells, control: 60 cells, dynamic groove (1 round): 61 cells, dynamic groove (2 rounds): 71 cells, dynamic groove‐to‐pit: 68 cells. Mean with SD. D) Effect of 50 nm Jasplakinolide (J) on the formation of focal adhesions on dynamic topographies (grooves of 90 µm wide). Mean with SD, n ≥ 4 analyzed samples and n > 40 cells analyzed per condition. A Fisher exact test (5% confidence interval) showed no significant differences of the number of FAs formed on the dynamic concave part between the dynamic grooves (2 rounds) and dynamic groove‐to‐pit samples, as well as between dynamic grooves (2 rounds) and dynamic grooves (2 rounds) + Jasplakinolide conditions. E) Cartoon showing focal adhesion formation on dynamic hydrogels.

Cells adhere to their ECM or underlying culture substrate by forming focal adhesions (FAs), which also allows them to transmit environmental mechanical stimuli intracellularly. Earlier, we observed that cells cultured in the presence of dynamic grooves (2 rounds) started to form protrusions and had lower solidity (Figure 2E), which point to changes in the cell adhesion dynamics. To better understand how cell adhesion is affected by the dynamic topographies, we more closely examined the formation and localization of focal adhesions (FAs). Before actuation, fibroblasts formed mature FAs, as indicated by vinculin staining (**Figure** **3**A). Surprisingly, despite minor changes in the overall cell morphology (Figure 2), hardly any mature FAs were present after one round of actuation (Figure 3A), whereas on flat controls FAs were still formed. Interestingly, after the second round of actuation, more FAs were formed than after one round of actuation without a significant change in FA area (Figure 3A,B; Figure S16, Supporting Information). It is worth noting that, during the second round of actuation, surface topographies were induced at the exact same location as during the first round of actuation. We hypothesized that the first round of dynamic topographies “conditioned” the cells to topographical changes, priming them to form new anchoring points to the substrate in response to future microenvironmental changes. If this was true, then there should be a difference between FA formation on parts of the substrate that underwent dynamic topographical change (i.e., the illuminated parts) versus parts that did not (i.e., the masked areas). Therefore, we analyzed the localization of the FAs after two rounds of dynamic topography and indeed found that the cells formed a small majority of their FAs (55%) on the dynamic, concave regions of the substrate than on the static part of the substrates (Figure 3C).

To investigate this further, we made use of the shape‐morphing capability of the photoresponsive SBS‐Sp‐pNIPAM hydrogels and subjected cells to concave grooves during the first round and, after overnight recovery, concave circular pits (dynamic groove‐to‐pit) during the second round. By changing the type of topographical pattern, we tested the shape‐dependent dynamic conditioning of the cells. We found that cells formed less FAs on the dynamic groove‐to‐pit substrate (29%) than on the dynamic groove–groove substrate (55%), while the number of FAs/cells formed at the static part was not changed (Figure 3C), confirming our hypothesis of dynamic topography‐specific conditioning of fibroblasts (Figure 3E).

Since the formation and maturation of focal adhesions are linked to the activity and stability of the actin filaments, we then asked whether pharmacological stabilization of the actin network could modulate the cells’ conditioning to dynamic topographies. Indeed, stimulating and stabilizing the actin network with Jasplakinolide resulted in the net formation of more focal adhesions on dynamic substrates than in control cells (17 vs 26 FA/cell) (Figure 3D). Additionally, cells treated with Jasplakinolide almost doubled focal adhesion formation on the dynamic concave part of the substrate than untreated cells (16 vs 9 FA/cell), suggesting that stimulating the assembly of the actin cytoskeleton better prepares the cell for future changes in the microenvironment.

**Figure 4:**
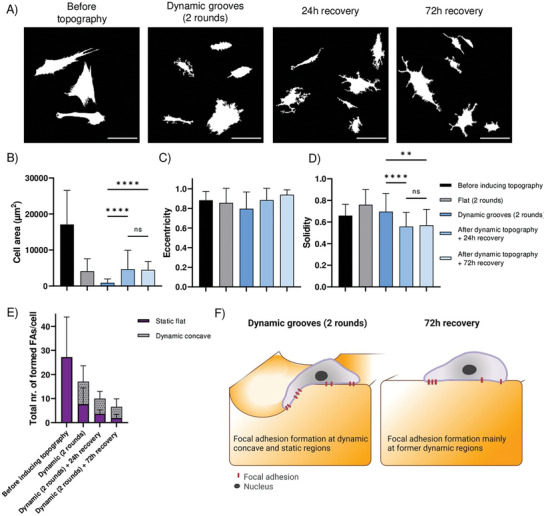
Cell recovery after inducing dynamic topographies (grooves, 2 rounds). A) Representative cell shapes on indicated topographies. Scale bar is 100 µm. B) Cell area. C) Cell eccentricity. D) Solidity. Mean with SD, n ≥ 23 analyzed cells. One‐way ANOVA. The Asterisk indicates statistically significant differences (^*^ *p* ≤ 0.05, ^**^ *p* ≤ 0.01, ^***^ *p* ≤ 0.001, ^****^ *p* ≤ 0.0001, ns not significant). E) Focal adhesion formation per cell per gel region. Mean with SD, n ≥ 5 analyzed samples with ≥ 22 cells per condition. F) Cartoon showing focal adhesion formation after cell recovery on dynamic hydrogels.

Next, we examined whether this conditioning to dynamic topographies is temporally maintained by the fibroblasts. To do so, we subjected the cells to two rounds of actuation (5 h, 90 µm wide grooves) and let them recover for 24 and 72 h (Movie S2, Supporting Information). Before topography was induced, fibroblasts had a stretched morphology, which was changed to a smaller and more elongated shape after 2 rounds of dynamic topographies. After 24 h of surface recovery, cells remained more elongated but started to form protrusions (**Figure** **4**A). Eventually, after 72 h recovery cells gained a starlike morphology, resembling cell morphology before substrate actuation (Figure 4A). The cells were able to recover their cell area to the same size as the cells that were cultured for the same duration on flat substrates, but not to the level prior to the induction of dynamic topographies (Figure 4B). Additionally, cells became more spread out after 72 h, comparable to cells before topographical conditioning (Figure 4A,D), although cell eccentricity was not altered significantly (Figure 4C). We noted that the amount of FA generally decreased over time in all conditions, consistent with a previous report on fibroblasts cultured on stiff hydrogels, where, on substrates stiffer than our hydrogels (2 MPa), less FA formed, although the formed FA were bigger and more mature.^[^ ^54^, ^55^ ^]^ Since fibroblasts showed little migration on the dynamic topographies (Movie S2, Supporting Information), we investigated location‐dependent FA‐formation after 24 and 72 h of surface actuation. Interestingly, most of the FAs were still formed at the former dynamic concave parts of the substrate (63% and 71% of total FA formation after 24 and 72 h, respectively) (Figure 4E,F; Figure S17, Supporting Information). This finding at the FA level, together with the data of dynamic topographical conditioning, raises the idea that, aided by local regulation of focal adhesions, fibroblasts can remember previous dynamic mechanical events.

### 2.4 Dynamic Topographies Affect Nuclear Morphology and Chromatin Organization

**Figure 5:**
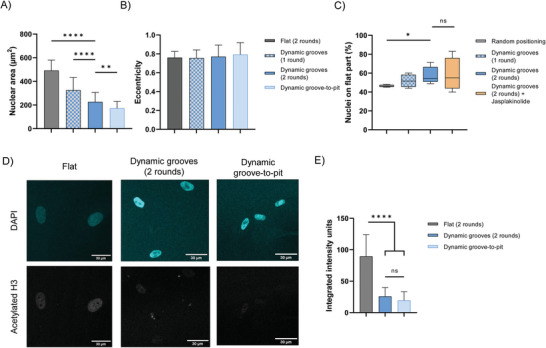
Influence of dynamic topographies on nuclear morphology and epigenetic modifications. A) Nuclear area. B) Nuclear eccentricity. C) Quantification of nuclei that are located on the flat static part of the hydrogel. D) Confocal images of nuclei (DAPI) and histone acetylation (AcH3) of cells cultured on flat or dynamic topographies (90 µm grooves or 90 µm wide concave pits). Scale bar is 30 µm. E) AcH3 intensity was quantified based on immunostaining. Mean with SD, with > 23 nuclei analyzed per sample. One‐way ANOVA, asterisk indicates statistically significant differences (^*^ *p* ≤ 0.05, ^**^ *p* ≤ 0.01, ^***^ *p* ≤ 0.001, ^****^ *p* ≤ 0.0001, ns not significant).

Inside the cell, FAs are connected to the actin cytoskeleton, which is also in direct contact with the nucleus. As a result, extracellular mechanical stimuli can affect the nucleus via transmitted forces.^[^ ^56^, ^57^ ^]^ Therefore, we sought to understand how dynamic topographical changes can influence nuclear morphology. We observed that the nuclear area was significantly smaller on the dynamic substrate after one and two rounds of dynamic 90 µm wide grooves (**Figure** **5**A) with no significant change in nuclear eccentricity (Figure 5B). Reconfiguring the geometry of the topography (dynamic groove‐to‐pit) resulted in even smaller nuclei than on the dynamic grooved substrate (2 rounds). Additionally, the nuclei showed no clear orientation with the induced topography (Figure S18, Supporting Information). Yet, we observed that cells tended to position their nuclei at the static, flat regions, away from the dynamic topographies (Figure 5C), which is significantly different from random positioning. The repositioning of the nucleus could already be observed after one round of actuation, although the difference is not statistically significant from random positioning and was enhanced after two rounds. Furthermore, stabilizing the actin cytoskeleton with Jasplakinolide did not further enhance the nucleus repositioning effect (Figure 5C).

Forces acting on the nucleus not only change nuclear morphology, but can also affect chromatin condensation and epigenetic modifications, such as histone acetylation. To investigate the effect of dynamic topographies on chromatin condensation, we stained the nuclei with DAPI and immunolabeled acetylated histone H3 proteins (Figure 5D). The fluorescence signal of acetylated histone was decreased in cells cultured on dynamic topographies (Figure 5E). Interestingly, the type of topography (anisotropic or isotropic pattern) did not seem to affect histone acetylation levels. Together, these data indicate that fibroblasts on dynamic topographies compact their genetic material, resulting in smaller nuclei and more condensed chromatin.

## 3 Discussion

In this study, we introduced a new dynamic hydrogel platform (SBS‐Sp‐pNIPAM) to examine the effect of recurring and reconfiguring topographies on fibroblast behavior. The presented dynamic topographical cell culture substrate does not suffer from concomitant changes in material properties (roughness, stiffness, and strain) upon illumination. Previously reported light‐responsive materials show a significant increase in roughness upon actuation,^[^ ^42^ ^]^ which is disadvantageous as cells are known to respond to nanometer‐scale changes in surface topography.^[^ ^58^, ^59^, ^60^ ^]^ Moreover, our culture platform allows material actuation that can be performed in the presence of medium in a standard incubator, which is not possible with previously reported azobenzene‐based systems that require a sophisticated dry‐confocal‐based illumination set‐up.^[^ ^40^, ^42^ ^]^ The fluorescence signal of the protonated merocyanine allows easy demarcation of the dynamic regions of the substrate, where isomerization to the spiropyran form takes place. In the presented work, isomerization to the spiropyran was shown to be possible for three successive rounds, and previous research demonstrated that at least four illumination cycles are also possible.^[^ ^46^ ^]^ The isomerization to the spiropyran form changes the hydrophilicity of the illuminated areas of the hydrogel, and because of the SBS topcoat, the water present in the hydrogel can only migrate to the nonilluminated areas, ultimately causing local hydrogel shrinkage. Consequently, the depth of the induced topography can be tuned by varying the width of the exposed area. Moreover, by decreasing the crosslinker density, deeper topographies can be induced. Thus, by a careful design of the hydrogel mixture, one can control the depth of the induced topographies. With our current setup, features < 50 µm result in overlap of shrunk areas, possibly through light scattering events. However, this challenge can be overcome by redesigning the mask and increasing the spacing between features. Despite this current limitation in feature resolution, we were able to induce topographies at the micrometer scale, which are almost two orders of magnitude larger than what has been achievable using photoresponsive liquid crystal polymer networks (tens of nm).^[^ ^45^ ^]^ Surface topographies could be induced in 15 min, which is slower than strain‐responsive reorientation of PDMS grooves (1 min),^[^ ^37^ ^]^ or the light‐induced reshaping of azopolymer‐based nanopillars (< 1 min),^[^ ^41^ ^]^ but comparable with light‐responsive azobenzene‐based materials (7 min).^[^ ^42^ ^]^

In principle, one can also use other stimuli‐driven dynamic substrates, such as temperature‐ or strain‐responsive materials.^[^ ^6^ ^]^ However, thermally activated shape‐memory polymers only allows non‐reversible switching between two predetermined topographies using reprogramming and requires high temperatures.^[^ ^36^, ^61^ ^]^ On the other hand, strain‐responsive PDMS systems have strong limitations in the design flexibility and variability of topographical patterns that can be obtained.^[^ ^37^, ^38^ ^]^ Our photoresponsive SBS‐Sp‐pNIPAM substrates offer a variety of topographies whose features can be flexibly chosen through the mask design and that can be induced by using non‐cytotoxic exposure to blue light, overcoming the limitation of other stimuli‐responsive materials. We note that, although light exposure for prolonged periods (2 × 5 h) in our setup did not affect cell viability nor induce DNA damage, it is possible that longer illumination might cause phototoxicity^[^ ^62^ ^]^ and should therefore always be pre‐tested. With our current approach, the SBS‐Sp‐pNIPAM hydrogel is relatively stiff (350 kPa) compared to the mechanical properties of soft tissues. It will be interesting in the future to explore the tunability of the hydrogel mechanical properties to dissect the roles of dynamic topography sensing and stiffness.^[^ ^63^, ^64^ ^]^

We showed a biological application of the presented dynamic topographical substrate as a way to provoke a mechanobiological response in fibroblasts. The applied topography (90 µm wide anisotropic grooves, κ = 3 mm^−1^) can be found in vivo as small vasculature (e.g., alveoli have d = 75–300 µm), gut villi, but also in the shape of small wounds (nano to millimeter scale) and the anisotropic organization of ECM protein fibers.^[^ ^8^, ^28^ ^]^ After two rounds of topographical actuation, fibroblasts displayed a smaller spread area on dynamic topographies than on the flat substrates, in accordance with previous literature using static topographies.^[^ ^1^, ^65^ ^]^ Surprisingly, we did not observe alignment of cells with the introduced anisotropic topography or an increase in cell elongation after one and two rounds of topography change. This is in contrast to previous studies that report fibroblast alignment on static topographies.^[^ ^58^, ^66^, ^67^, ^68^ ^]^ For example, murine fibroblasts were reported to align within 48 h on grooved PDMS membranes (10, 20, and 40 µm wide, all 3 µm high)^[^ ^58^ ^]^ and human stromal fibroblasts were shown to align within 24 h with static topographies on silicon chips, although the depth of the topography was found to be crucial in guiding cell alignment (< 550 nm, cell alignment was not considered to be significant).^[^ ^68^ ^]^ More recently, live‐cell imaging of myoblasts on strain‐responsive PDMS grooves (2.5 µm wide, 0.32 µm high grooves) showed that the cells started to align with the new topography after 4 h, and after 6–8 h cells did not further change their alignment.^[^ ^37^ ^]^ Another study reported alignment of murine fibroblasts 5 h after inscription of the pattern (3 µm wide, ca. 400 nm high grooves).^[^ ^40^ ^]^ In our dynamic topography setup, after 5 h of topography inscription we did not obtain the same orientation response as reported previously within the same time span. This could be attributed to the much wider applied anisotropic patterns in our approach (90 µm) than the width of topographies used in the beforementioned works. Indeed, previous studies using fibronectin micropatterned lines revealed that cell alignment of myofibroblasts decreases with wider patterns.^[^ ^29^, ^69^ ^]^ Future dedicated studies should therefore be directed to systematically vary and investigate the influence of (dynamic) topography dimensions on cell response.

**Figure 6:**
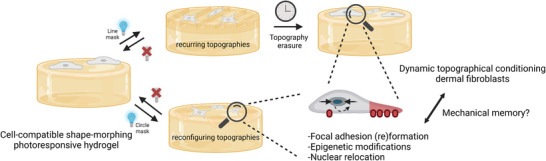
Shape‐morphing photoresponsive hydrogels reveal dynamic topographical conditioning of dermal fibroblasts, mediated by focal adhesion (re)formation, epigenetic regulation, and nuclear repositioning, allowing cells to remember their previous topographic events.

Our approach allows us to, for the first time, subject fibroblasts to multiple rounds of shape‐morphing topographies. Strikingly, focal adhesions were found to disappear after one round of actuation and reform after the second round of actuation. We hypothesize that, by decreasing the amount of focal adhesion sites, the cells are better primed to adapt to further topographical changes in the environment, and substrate deformation will affect the cells to a lower extent. This has an interesting potential in vivo significance, for example in conditioning dermal fibroblasts to maintain skin homeostasis or promote wound healing.^[^ ^70^ ^]^ Consistent with this hypothesis, we found that, after the second round of recurring topography change, fibroblasts more readily formed more FAs on the dynamic regions of the hydrogel. Even after 72 h recovery, most FAs were formed at the former dynamic regions of the hydrogel. In contrast, when the shape of the topography was reconfigured, fibroblasts formed less FAs on the dynamic groove‐pit substrate than on the dynamic groove–groove substrate. Uncovering the mechanistic basis of this intriguing difference between FA formation on recurring and reconfiguring substrates will therefore be exciting. For instance, it may be caused by a mechanically induced conformational change in the FA machinery exposed to dynamic regions (e.g., vinculin) during the first round, which in turn induces the FA complexes to be locally pre‐assembled on the groove–groove situation and not on the groove‐to‐pit substrates. Our data suggest that cells can remember mechanical events occurring during topography change through regulation of the focal adhesions at the cell‐substrate interface. This is in line with current thoughts about the protective role of FA pre‐adaptation during mechanical disruption, enabling cells to counteract and withstand future mechanical events via feedforward mechanisms, providing a so‐called “insurance mechanism”.^[^ ^71^ ^]^ Besides, this is reminiscent of the recently proposed concept of “mechanical memory”, which allows cells to remember the stiffness of hydrogel on which they had been previously seeded.^[^ ^72^ ^]^ Our work further points to the intriguing possibility that such “mechanical memory” may also be induced by other physical cues (i.e., dynamic topography) (**Figure** **6**). Future work, for example combining super‐resolution microscopy and volumetric electron microscopy, will provide a more detailed and comprehensive understanding of the complex FA function in establishing FA‐mediated mechanical memory. Moreover, the effect of dynamic reconfigurations on exerted cell forces could be measured using traction force microscopy, as well as the force originating from dynamic reconfigurations sensed by the cell using molecular tension sensors.^[^ ^73^, ^74^, ^75^ ^]^

Work over the last 10 years established the fact that cells can transmit and transduce mechanical signals from the environment to the nucleus. On the dynamic topographies presented here, fibroblasts displayed smaller nuclei and positioned their nuclei mostly on the static parts of the substrate (Figure 6). This may indicate that cells condense the chromatin and try to minimize the impact of microenvironmental topographical changes on the DNA.^[^ ^76^, ^77^ ^]^ Furthermore, we observe a decrease in histone acetylation for cells cultured on dynamic topographies, which is consistent with previous studies on static topographies,^[^ ^58^ ^]^ as well as on in situ micropatterned azopolymer films.^[^ ^78^ ^]^ By lowering acetylation, the chromatin is folded more tightly, giving the cells another way to maintain genome stability and protect against unwanted deformations. In line with this, Luciano et al. reported that static concave curvature promotes chromatin condensation and smaller nuclei in epithelial cells.^[^ ^15^ ^]^ While it has been shown that inhibiting the actin–myosin machinery impairs the epigenetic effect of mouse fibroblasts cultured on microgrooves,^[^ ^58^ ^]^ and that uniaxial stress enhances the linkage between the nuclear envelope and the actin cytoskeleton,^[^ ^79^ ^]^ our work stresses the need to explore how the epigenetic machinery is exactly altered in the presence of dynamic physical events.

## 4 Conclusion

Using shape‐morphing cell‐culture substrates whose topography can be changed on demand upon blue‐light exposure, fibroblast response to dynamic topographies was investigated. The substrate consists of an SBS‐coated spiropyran‐based pNIPAM hydrogel, whose topography can be easily and repeatedly modulated by masked illumination and erased in the dark, in the presence of cells, without affecting cell viability or inducing DNA damage. Fibroblasts were revealed to exhibit dynamic topographical conditioning to the induced patterns by reorganizing their cell shape, FA formation, and nuclear matter, possibly to allow them to prepare for future topographical events. The FA reorganization was maintained after removal of the surface topography, suggesting that fibroblasts retain memory of previous topographical events. In conclusion, the presented approach is an attractive way to study cell response to multiple user‐defined topographical and geometrical changes, allowing better recapitulation of the dynamic cellular microenvironment in vivo. Ultimately, this can help in a better understanding of dynamic biomechanical processes, as they occur for example during wound healing or disease progression, and the underlying (nuclear) mechanotransductive pathways that regulate cell response.

## 5 Experimental Section

### Materials

All materials were obtained from commercial sources, unless stated otherwise. 6‐(1′,3′,3′‐trimethylspiro[chromene‐2,2′‐indole]−8‐yl)oxyhexyl acrylate (spiropyran) was produced by SyMO‐Chem BV, Eindhoven. The UV photoinitiator 2‐hydroxy‐2‐methyl‐1‐phenyl‐1‐propanone (Darocur 1173), and anhydrous 1,4‐dioxane, acrylic acid, N‐isopropylacrylamide (NIPAM), N,N′‐methylenebis(acrylamide) (MBIS), 1H,1H,2H,2H‐perfluorodecyltriethoxysilane and 3‐(trimethoxysilyl)propyl methacrylate were purchased from Sigma‐Aldrich and used without further purification.

### Substrate Preparation

Glass substrates (1.5 cm x 1.5 cm) were cleaned in 2‐propanol by sonication (15 min) and dried with a flow of nitrogen. Afterward, the glass slides were treated in a UV−ozone photoreactor (UV Products, PR‐100, 20 min). The surface of the glass substrates was modified by spin‐coating a 3‐(trimethoxysilyl)‐propyl methacrylate solution (0.5% v/v solution in a 1:1 water−2‐propanol mixture) or a 1H,1H,2H,2H‐perfluorodecyltriethoxysilane solution (1% v/v solution in ethanol) on the cleaned glass substrate for 45 s at 3000 rpm. Subsequently, the glass slides were cured at 100°C for ≈10 min.

### Hydrogel Preparation

Hydrogels were produced as described previously^[^ ^46^ ^]^ using a 2:1 dioxane‐water monomer cocktail (0.44 mg µL^−1^), consisting of 87 mol% NIPAM, 10 mol% acrylic acid, 1 mol% MBIS, 1 mol% spiropyran and 1 mol% Darocur 1173.

### Substrate‐Constrained Hydrogel Films

Thin films of the hydrogels were prepared and attached to a propyl methacrylate functionalized glass slide, using a handmade cell with ≈50 µm spacing. The cell consisted of a propyl methacrylate functionalized bottom glass slide and an upper glass slide functionalized with the 1H,1H,2H,2H‐perfluorodecyltriethoxysilane solution. The cell was capillary filled with the hydrogel monomer mixture and exposed to UV light (5 min, 3.44 mW cm^−2^, J110619 Analytik Jena). After polymerization, the upper glass slide was removed and the dioxane was allowed to evaporate (30 min). The hydrogels were washed 3x with demineralized water and swollen overnight in the dark in demineralized water until use. Next, hydrogel films were spin‐coated with a styrene‐butadiene‐styrene (SBS, 100 mg mL^−1^ in toluene, D1102, Kraton) layer using a Karl Süss RC‐8 spin coater at 500 rpm for 120 s. Samples were dried for 10 min at room temperature and stored in demineralized water until use.

### Illumination: Inducing Topographical Patterns

An in‐house illumination holder was made to illuminate a 12‐well culture plate from the bottom. In this holder, soda‐lime photomasks with chromium‐designed patterns (designed in KLayout v 0.26.9 and produced by Techno‐Mask, Eindhoven) and two lamps fit. Light illumination was performed using collimated LEDs (455 nm, 700 mA, M455L4‐C4, Thorlabs) connected to a LED controller (M00647125, Thorlabs). For cell studies, constructs were illuminated in a humidified incubator at 37°C.

### UV–vis Spectroscopy

The photoresponse of SBS‐coated hydrogel constructs was measured using UV–vis spectroscopy (Shimadzu UV‐3102 PC UV–vis–NIR scanning spectrophotometer). Constructs were soaked in a basic cell culture medium without phenol red for 24 h (DMEM 31053‐028, Thermofisher). Next, the photoresponse and recovery time of the gel was determined at room temperature.

### Nanoindentation

A Piuma nanoindenter was used to measure the stiffness of the hydrogel constructs (n = 4) before and after 455 nm illumination. Each gel was indented at 6 or 7 randomly selected positions. Probes (P211070M, Optics11) with a tip radius of 21 µm and a spring constant of 0.16 N m^−1^ were used in PBS and at room temperature. Prior to illumination and/or indentation, constructs were exposed to UV light (20 min, 3.44 mW cm^−2^) for sterilization.

### Atomic Force Microscopy

Surface roughness was determined by measuring the surface of the gel (n = 3) at 3 randomly selected positions (20×20 µm each measurement) before and after illumination (455 nm, 15 min) using an atomic force microscope (Bruker Bioscope Catalyst mounted on a Leica DMi6000B inverted optical microscope) in contact mode with a SNL‐10 probe (tip radius 2 nm, Bruker). Surface roughness (Rq) was calculated using the NanoScope Analysis software v150.

### Optical Profilometry

Height profiles and 3D images of the (patterned) hydrogels were captured using an optical surface profiler (Sensofar Plµ 2300 with a 20x/0.45 NA Nikon objective). Data was processed using Plu Optical Imaging Profiler 2.41 software.

### Cell Culture and Seeding

Normal human dermal fibroblasts, nhDF (Lonza, CC‐2511), were grown in Dulbecco's Medium Eagle's Medium (DMEM 41966‐029, Thermofisher) supplemented with 10% fetal bovine serum (FBS; 758093, Serana) and 1% penicillin–streptomycin (15140163, Gibco). Cells were cultured in T‐25 cell flasks at 37°C in a humidified atmosphere with 5% CO2. Prior to cell seeding, hydrogel constructs were sterilized by 20 min UV exposure (λ = 254 nm) and coated with 10 µg mL^−1^ fibronectin in PBS by a 30 min incubation at room temperature (bovine plasma fibronectin, 1 mg mL^−1^, 33010–018, Invitrogen). Fibroblasts were dissociated using 0.05% trypsin/Ethylenediaminetetraacetic acid (EDTA) with phenol red (25300054, Gibco). A total of 22000 cells were seeded on each construct. After overnight adhesion, topographical patterns were induced as described above. All experiments were performed with cells until passage 15.

### Jasplakinolide Treatment

Cells were seeded on hydrogel constructs as described above. After overnight adhesion, Jasplakinolide in DMSO (2792, Tocris) was added to the medium and carefully mixed to reach a final concentration of 50 nm Jasplakinolide. After 15 min, topographical patterns were induced.

### Immunofluorescence Assay

Cells were fixed with 3.7% paraformaldehyde (formalin 37%; 104033.1000, Merck) for 15 min at room temperature, washed with PBS, permeabilized for 30 min with 0.5% Triton‐X‐100 (108643, Merck) in PBS and blocked for 15 min with 4% goat serum in 0.05% PBS‐Tween. Samples were labeled with the primary antibodies rabbit anti‐AcH3 (1:300, 06–599, Sigma–Aldrich), or mouse anti‐vinculin (1:600, V9131, Sigma–Aldrich), and incubated overnight at 4°C or for 1.5 h at room temperature. After washing with PBS‐Tween, samples were incubated with Phalloidin‐Atto 590 (1:54, 93042, Sigma–Aldrich) and the secondary antibodies goat anti‐mouse‐Alexa 647 (1:300, A21240, Molecular Probes) or goat anti‐rabbit‐Alexa 647 (1:500, A21244, Molecular Probes) for 1 h at room temperature. After washing with PBS, nuclei were stained using 4′,6‐diamidino‐2‐phenylindole dihydrochloride (D9542, Sigma–Aldrich).

### Image Acquisition and Experimental Data Analysis

Stained hydrogel constructs were imaged using a Leica TCS SP5 confocal microscope with a 20×, 0.7 NA objective. Z‐stacks were recorded at a 2 µm Z‐spacing. The signal of the protonated merocyanine was acquired by excitation at λ = 488 nm and detection of the emission at *λ* = 501–570 nm. CellProfiler pipelines were used to measure morphological parameters, such as object area, eccentricity, and orientation. For quantifying AcH3 levels, all samples were stained together and imaged with the same settings, and the integrated intensity was measured in CellProfiler. Focal adhesion images were processed and analyzed in ImageJ based on the protocol by Horzum et al.^[^ ^80^ ^]^ A TopHat filter was applied to remove pixel noise and enhance contrast. Only particles between 2 and 30 µm^2^ were analyzed, and identified objects were always examined by eye to confirm noise was not included in the analysis.

A custom‐made MATLAB script was used to determine the minimal distance between the center of an object (nucleus) and the center of the closest line pattern (90 µm wide lines). If this distance was between 0 and 45 µm, the object was identified to be on the flat region of the gel, if this was > 45 µm the object was classified to be on the dynamic, concave part of the gel. The output was always validated by eye.

### Statistical Analysis

Data were plotted as mean with standard deviation (SD). Statistical analyses were performed using GraphPad Prism 9. To test for significant differences in material roughness and stiffness between gels before and after light exposure, a paired *t*‐test was performed. To test for significant differences in the distribution of orientation data, a Kolmogorov–Smirnov test was performed. To compare multiple experimental conditions with each other, a one‐way ANOVA was performed with Tukey's post hoc analysis. For categorial focal adhesion data, a Fisher's exact test was performed. Differences were considered to be significant for *p*‐values ≤ 0.05.

Graphical representations were made with BioRender.com.

## Conflict of Interest

The authors declare no conflict of interest.

## Author Contributions

M.B. contributed in conceptualization, methodology, investigation, data analysis, visualization, and writing—original draft. A.S. contributed in conceptualization and methodology. I.J. contributed in investigation and data analysis. M.C.T. contributed in software. A.P.H.J.S. contributed in methodology and resources. C.M.S. contributed in conceptualization. C.V.C.B. contributed in supervision. N.A.K. contributed in conceptualization, supervision, funding acquisition, and writing—review & editing.

## Supporting information

Supporting Information

Supplemental Movie 1

Supplemental Movie 2

## Data Availability

The data that support the findings of this study are available from the corresponding author upon reasonable request.
