# Supplementary material for: Shape‐Morphing Photoresponsive Hydrogels Reveal Dynamic Topographical Conditioning of Fibroblasts
Source: Adv Sci (Weinh). 2023 Sep 23;10(31):2303136. doi: 10.1002/advs.202303136 (PMC10625123; doi:10.1002/advs.202303136)
Supplement: Supplementary file 1 — Supporting Information [file ADVS-10-2303136-s002.pdf]

ADVANCED SCIENCE
Open Access

Supporting Information

for *Adv. Sci.*, DOI 10.1002/advs.202303136

Shape-Morphing Photoresponsive Hydrogels Reveal Dynamic Topographical Conditioning of Fibroblasts

*Maaike Bril, Aref Saberi, Ignasi Jorba, Mark C. van Turnhout, Cecilia M. Sahlgren, Carlijn V.C. Bouten, Albert P.H.J. Schenning and Nicholas A. Kurniawan**

Supporting Information

Shape-morphing Photoresponsive Hydrogels Reveal Dynamic Topographical Conditioning of Fibroblasts

*Maaike Bril, Aref Saberi, Ignasi Jorba, Mark C. van Turnhout, Cecilia M. Sahlgren, Carlijn V.C. Bouten, Albert P.H.J. Schenning, Nicholas A. Kurniawan**

Materials and Methods

Measuring thickness of SBS layers: Glass slides were spin-coated with SBS in toluene according to the protocol described in the main text. Random scratches in the SBS layer were made using a razor blade, and the distance between the glass slide and SBS layer were measured using optical profilometry (Sensofar Plμ 2300 with a 20×, 0.45 NA Nikon objective). Data was processed using Plu Optical Imaging Profiler 2.41 software.

Rhodamine-fibronectin coating of SBS-coated pNIPAM gels: Hydrogel constructs were sterilized by 20 min UV exposure and coated with 10 µg/mL rhodamine-tagged fibronectin in PBS for ~60 min (bovine plasma fibronectin, FNR01, Cytoskeleton). Fluorescence signals were measured using a Leica TCS SP5 confocal microscope with a 10×, 0.4 NA objective.

Live/Dead viability assay: nhDF cells were seeded on hydrogel constructs and topography was induced as described in the main text. Prior to, or 1h after inducing topography, cells were washed with PBS and stained with CalceinAM (1.5 µM, 17783, Merck Life Science NV) and propidium iodide (3 µM, P4864, Merck Life Science NV) in PBS. Samples were incubated at 37 °C in a humidified atmosphere with 5% CO2 for 25 min. After incubation, the staining solution was removed, and the samples were washed with PBS and stored in PBS at 37 °C to prevent the sample from drying out. Stained cells were imaged using a Leica TCS SP5 confocal microscope with a 10×, 0.4 NA objective.

Etoposide treatment: Cells were seeded on 10 µg/mL fibronectin-coated glass coverslips (d=10 mm). After 48h, medium was changed to medium containing 20 µM etoposide in DMSO (E1383, Sigma-Aldrich), medium with, or without DMSO. After 24h of incubation,

cells were fixed with 3.7% paraformaldehyde (formalin 37%; 104033.1000, Merck) for 15 min at room temperature. Cells were immunolabeled as described below.

gamma H2Ax immunostaining and image acquisition: Cells were fixed with 3.7% paraformaldehyde (formalin 37%; 104033.1000, Merck) for 15 min at room temperature, washed with PBS, permeabilized for 15 min with 0.5% Triton-X-100 (108643, Merck) in PBS and blocked for 20 min with 4% goat serum in 0.05% PBS-Tween. Samples were labeled with the primary antibody mouse anti-gamma H2AX (phospho S139) (1:300, ab22551, Abcam) and incubated overnight at 4 °C. After washing with PBS-Tween, samples were incubated with the secondary antibody goat anti-mouse-Alexa 647 (1:200, A21240, Molecular Probes) for 1h at room temperature. After washing with PBS, nuclei were stained using 4′,6-diamidino-2-phenylindole dihydrochloride (D9542, Sigma-Aldrich). Stained hydrogel constructs were imaged using a Leica TCS SP5 confocal microscope with a water immersion 63×, 1.2 NA objective. Z-stacks were recorded at a 0.25 µm Z-spacing.

Live-cell imaging. Brightfield images of fibroblasts on hydrogels were taken every 15 minutes using a CytoSMART Lux (Axion BioSystems) live-cell imaging system with a 5× objective that was placed inside a humidified incubator (37 °C, 5% CO_2).

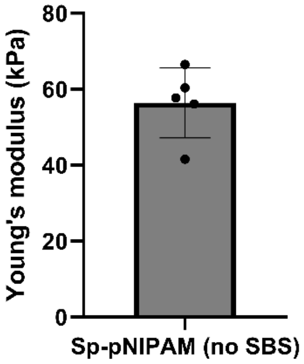

Figure S1. Stiffness of uncoated Sp-pNIPAM hydrogels, as measured with nanoindentation. Mean ± SD; each dot represents one hydrogel (n = 5 hydrogels). Each hydrogel was indented at 5 randomly selected positions and the averaged value was plotted.

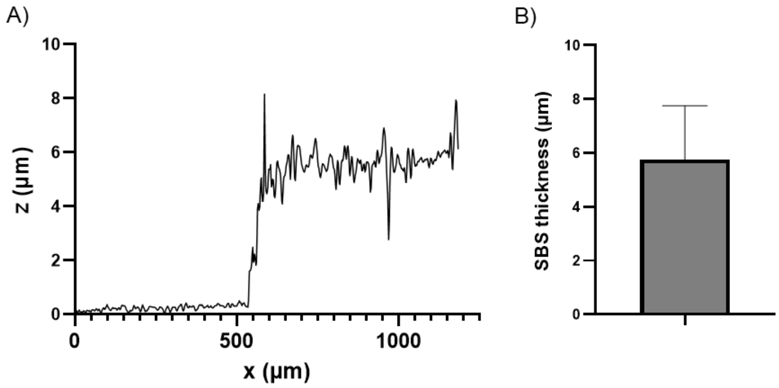

Figure S2. Thickness of the SBS layer measured using optical profilometry. A) Height profile. B) Measured thickness. Mean ± SD, n = 3 different samples measured at at least 3 random positions.

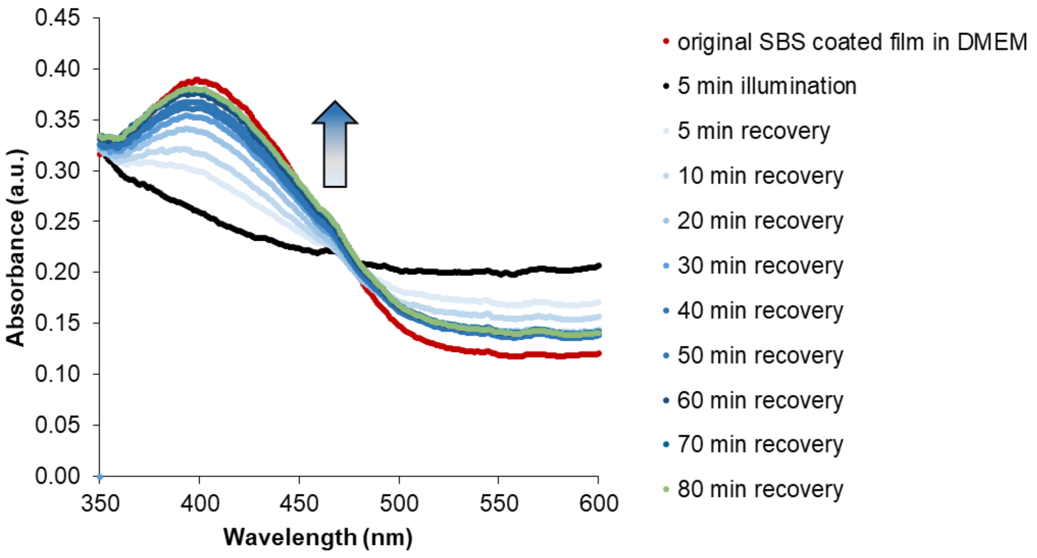

Figure S3. Representative UV-vis spectrum of SBS-Sp-pNIPAM hydrogel in cell culture medium. The spiropyran isomerizes spontaneously to the protonated merocyanine after illumination with blue light (455 nm).

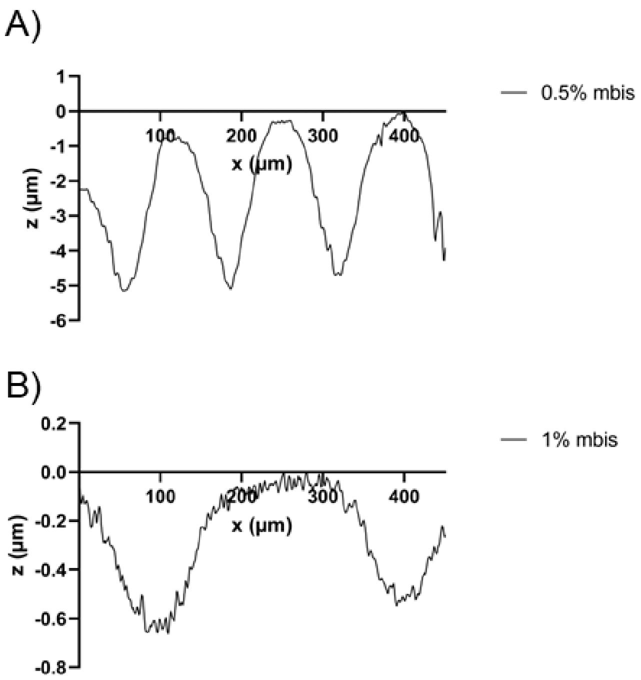

Figure S4. The effect of crosslinker density (methylene bisacrylamide, mbis) on profile generation after masked illumination (150 µm wide grooves, 15 min). A) 0.5% mbis SBS-Sp-pNIPAM hydrogel. B) 1% mbis SBS-Sp-pNIPAM hydrogel.

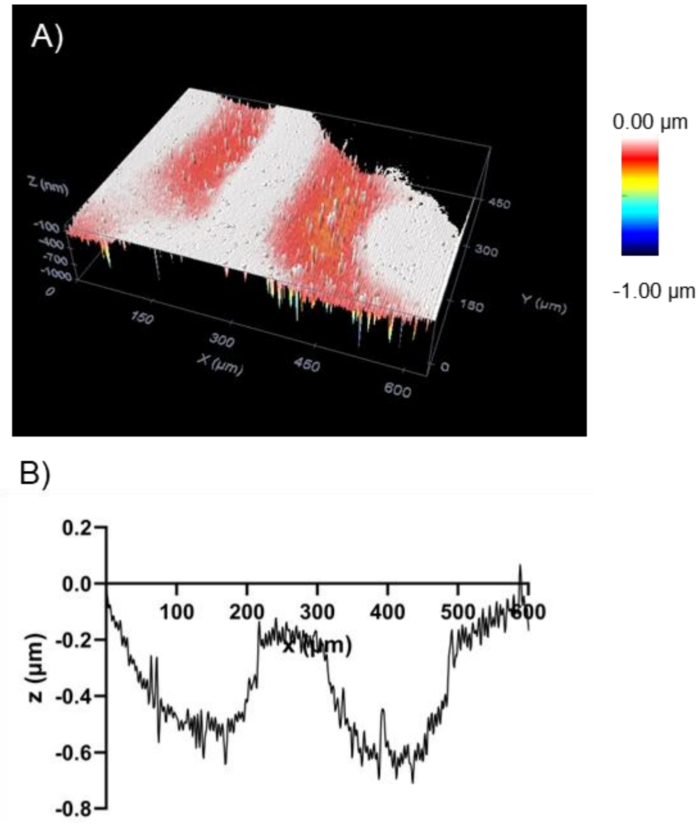

Figure S5. Inducing 30 μm wide grooves results in pattern overlap in SBS-Sp-pNIPAM hydrogels. A) 3D representation. B) Surface profile.

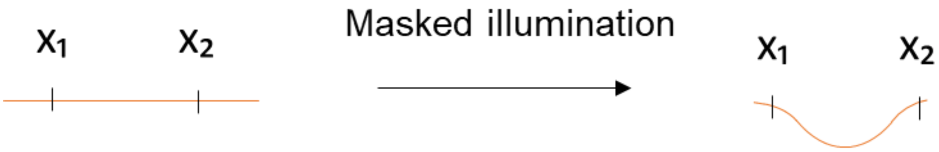

Figure S6. The surface contour length between two arbitrary chosen points was measured before and after actuation.

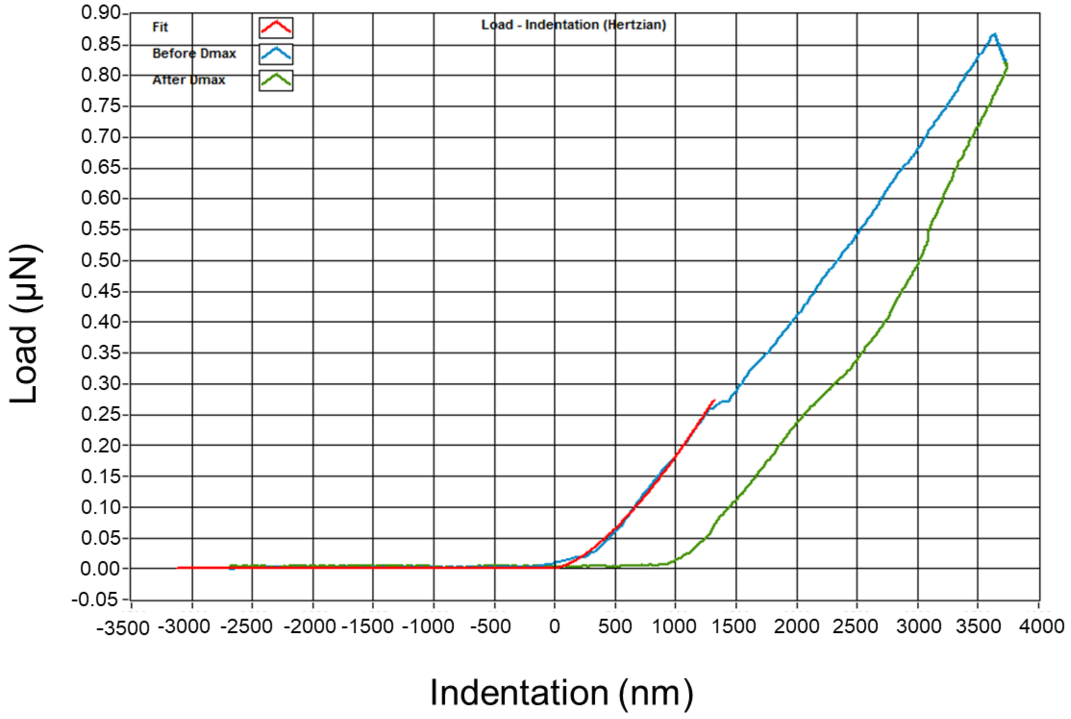

Figure S7. Representative nanoindentation profile of SBS-Sp-pNIPAM hydrogels after illumination.

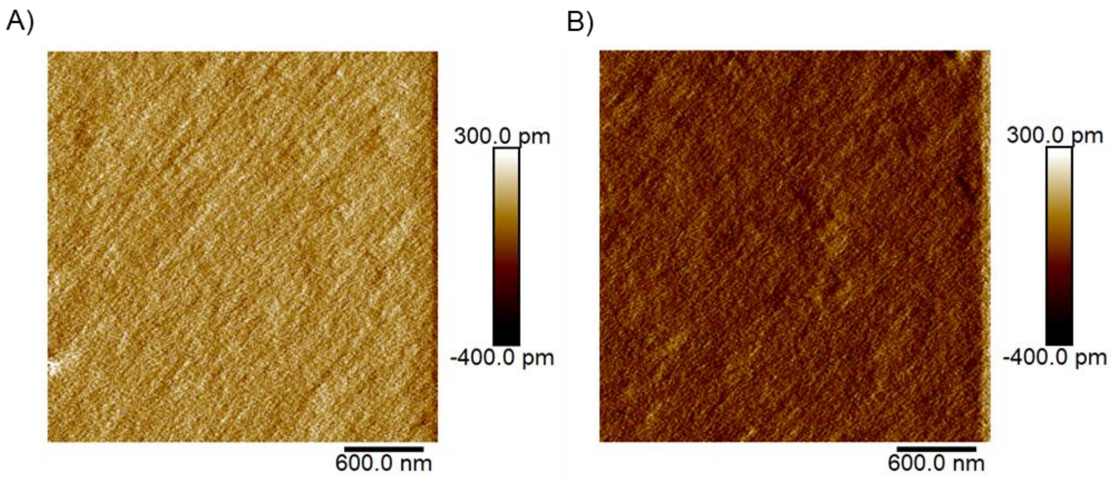

Figure S8. Surface roughness of SBS-Sp-pNIPAM hydrogels measured with AFM in contact scanning mode. A) Before illumination. B) After 15 min illumination (455 nm).

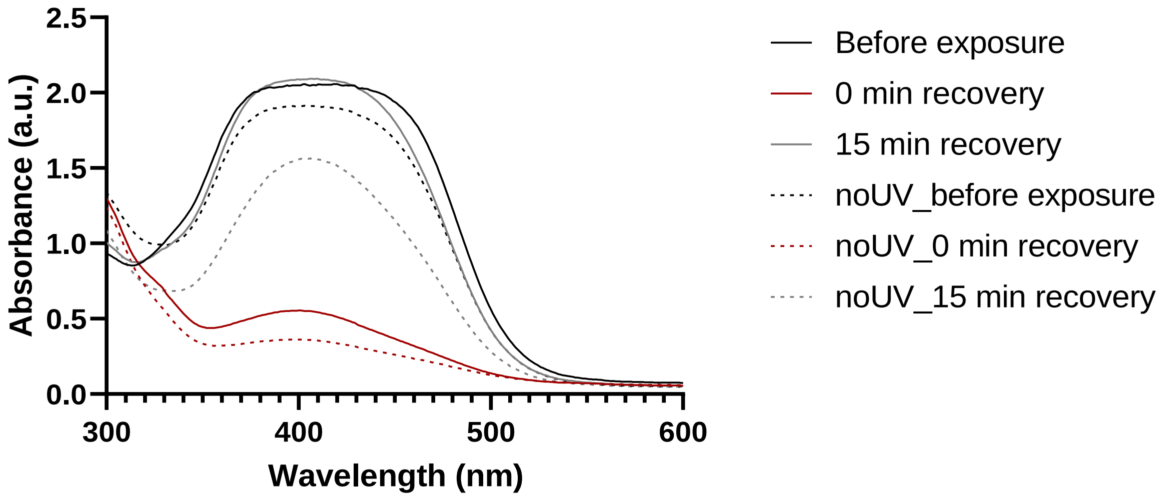

Figure S9. UV-vis spectrum of SBS-Sp-pNIPAM gels after 20 min UV-sterilization (254 nm) (solid lines), or without UV-illumination (dashed lines). Gels were soaked in demi water during UV-sterilization and blue light illumination (5 min., 455 nm).

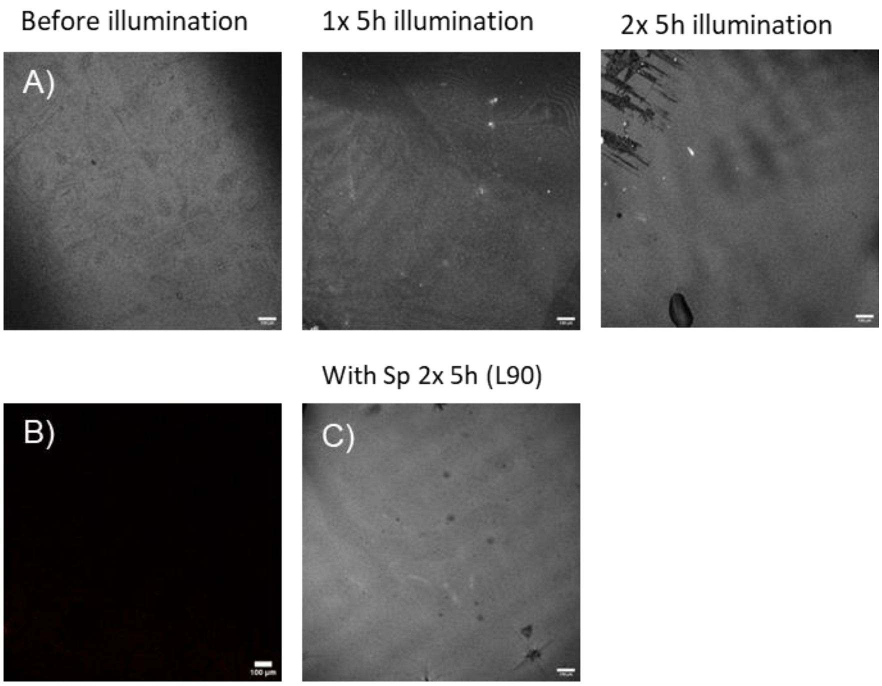

Figure S10. Surface coating of SBS-pNIPAM hydrogels A) A homogenous rhodamine-fibronectin coating (10 µg/mL in PBS) is present on static SBS-pNIPAM hydrogels, both before and after illumination (1 or 2 rounds). B) As a negative control, SBS-pNIPAM hydrogels were coated with PBS. C) Presence of rhodamine-fibronectin after 2 rounds of actuation (5h, L90) on dynamic SBS-Sp-pNIPAM hydrogels. Scale bar is 100 µm.

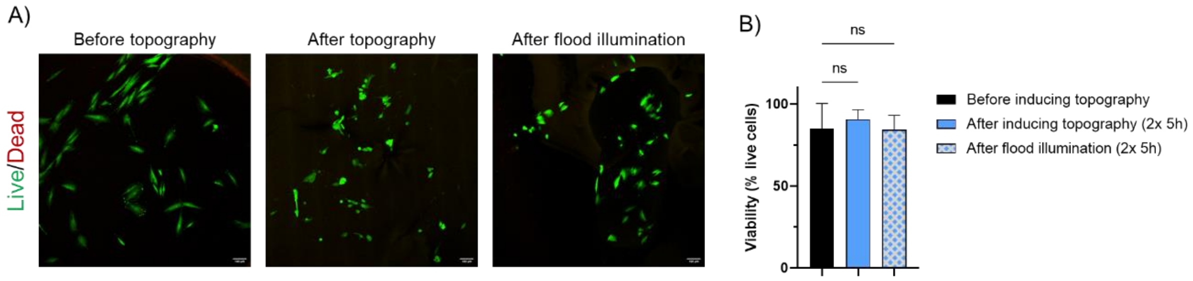

Figure S11. Cell viability on dynamic SBS-Sp-pNIPAM gels. A) Representative live/dead viability image before and after inducing grooved topographies. Live cells (nhDF) are stained with CalceinAM (green), dead cells with propidium iodide (red), scalebar = 100 µm. B) Cell viability (% viable cells) before and after inducing grooved topographies. Mean with SD, n ≥ 4 samples. Ordinary one-way ANOVA, ns = not significant ($p > 0.05$).

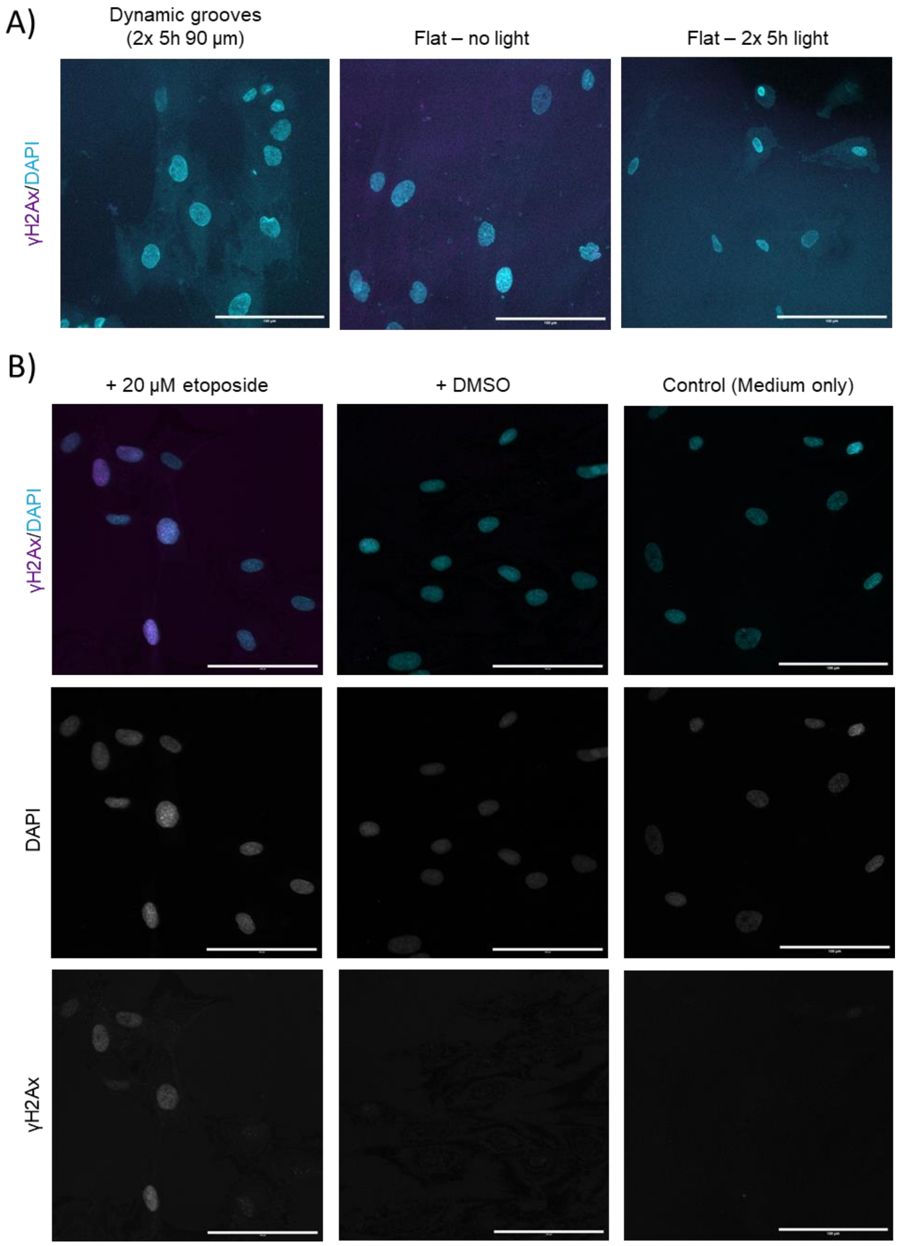

Figure S12. Detection of DNA damage using immunofluorescence microscopy. Upon double-stranded DNA breaks, histone γH2Ax gets phosphorylated and thereby amplifies the DNA damage response signaling cascade. A) Representative confocal images of nhDF cultured on SBS-Sp-pNIPAM hydrogels. Nuclei are stained with DAPI (blue), and γH2Ax is immunolabeled (purple). B) Representative images of nhDF treated with 20 µM etoposide (24h), a known inducer of DNA double-stranded breaks. Scale bar is 100 µm.

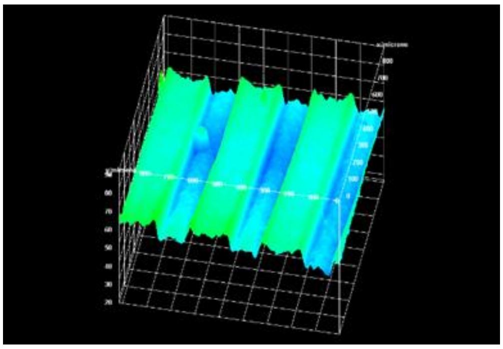

Figure S13. 3D representation of SBS-Sp-pNIPAM hydrogels after 15 min illumination with a 150 µm open line mask in an humidified atmosphere (37 °C). The fluorescence signal of the pronated merocyanine was detected using confocal microscopy, and z-stacks were converted into a 3D surface plot using ImageJ.

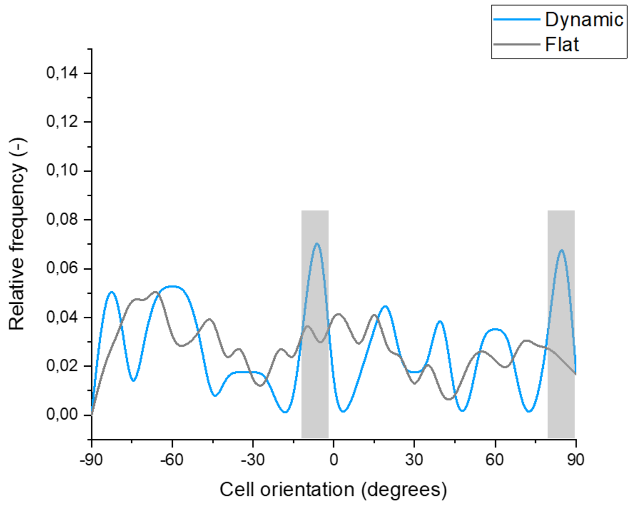

Figure S14. Cell orientation on flat and dynamic hydrogel constructs, where 0° indicates parallel alignment and 90° indicates perpendicular alignment with the induced grooves (shaded areas). A Kolmogorov-Smirnov test showed a significant difference in the distribution of the orientation data between dynamic and flat samples.

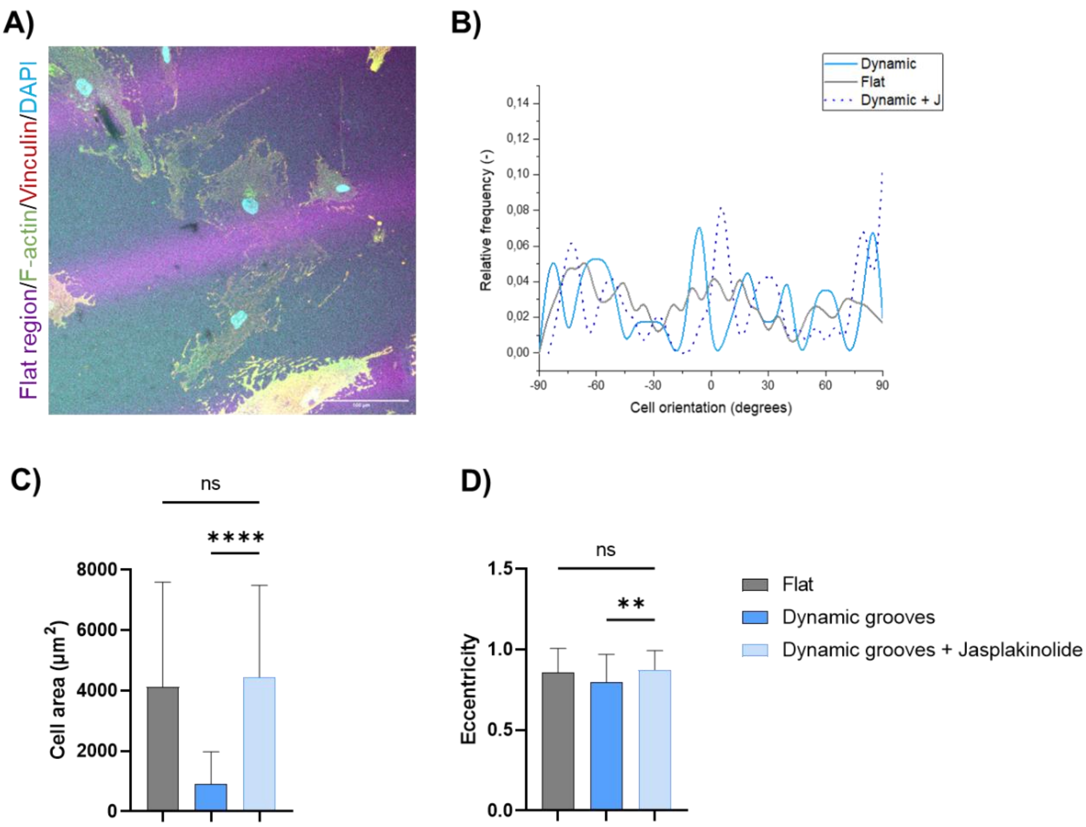

Figure S15. Cells on dynamic topographies (90 µm wide grooves, 2 rounds (5h)) in the presence of Jasplakinolide. A) representative confocal image of nhDF treated with Jasplakinolide (50 nM) on dynamic topographies. Cells were stained for F-actin (green), vinculin (red), and nuclei (blue). The flat static region of the gel is shown in magenta. Scale bar is 100 µm. B) Cell orientation on photoresponsive hydrogels. J = Jasplakinolide. C) Quantification of cell area. D) Cell eccentricity. Mean with SD, $n \geq 47$ cells. Ordinary one-way ANOVA. Ns = not significant, ** $p = 0.0038$, **** $p < 0.0001$.

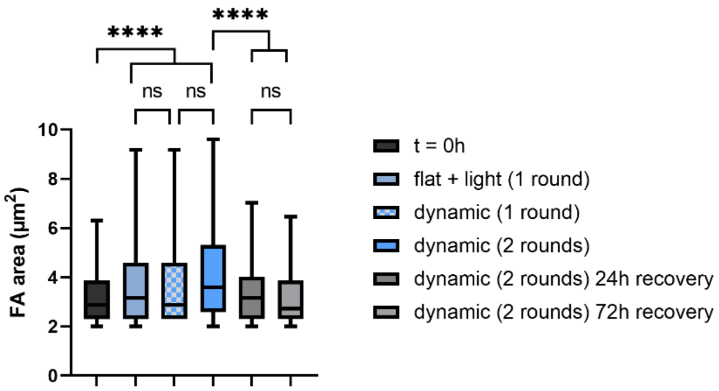

Figure S16. Focal adhesion area (µm^2) on SBS-Sp-pNIPAM hydrogels, before (t = 0h) and after introducing dynamic topographies (90 µm wide grooves). Box plot depicting minimum and maximum values, n ≥ 83 FAs. Ordinary one-way ANOVA with Tukey's multiple comparisons test. Ns = not significant, **** $p < 0.0001$.

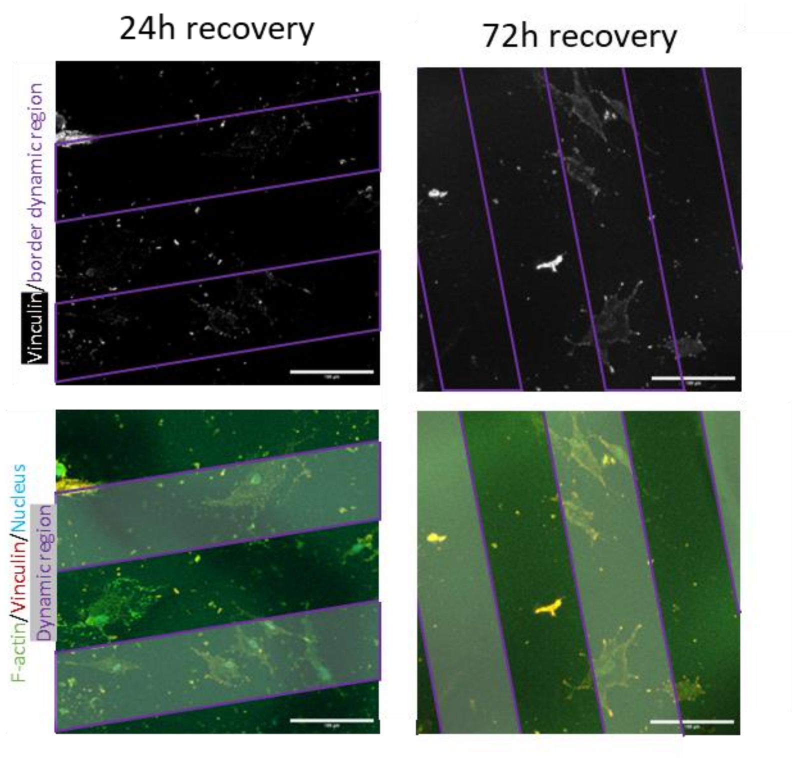

Figure S17. Focal adhesion formation of nhDFs 24h and 48h after inducing dynamic topographies (2 rounds of 90 µm wide grooves, 5h) on SBS-Sp-pNIPAM hydrogels. 24h or 48h after surface recovery, cells were fixed and stained for F-actin (green), vinculin (red), and nuclei (blue). The dynamic regions of the gel are shown as shaded areas. Scale bar = 100 µm.

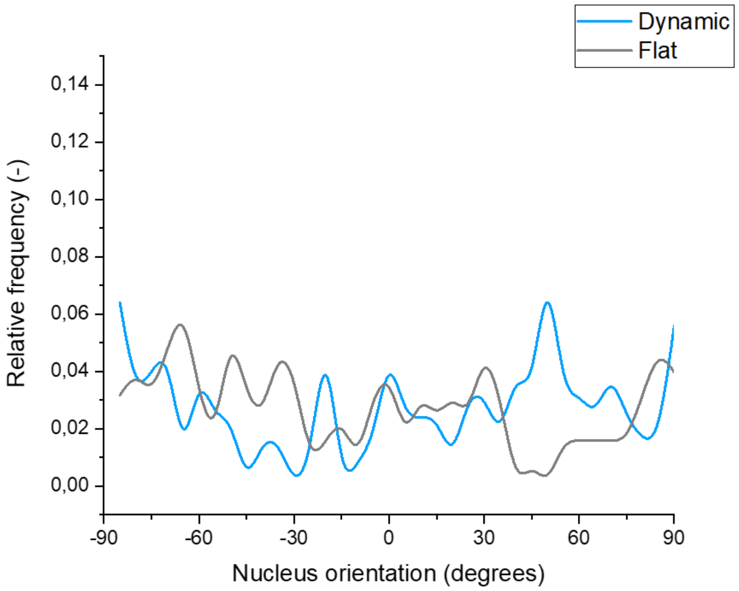

Figure S18. Orientation of the nucleus on flat and dynamic hydrogel constructs. nhDFs were subjected to two rounds of actuation (90 µm wide grooves, 2x 5h).

Table S1. Surface distances were calculated before and after masked light actuation (90 µm wide lines) using optical profilometry data. The surface distance after actuation was calculated by using the Pythagorean theorem and by using the equation that corresponds to a fitted parabola on the data.

Sample ID	Surface distance before actuation (µm)	Surface distance after actuation (µm) using Pythagorean theorem	Fold change before and after actuation	Surface distance after actuation (µm) using fitted parabola	Fold change before and after actuation
90_R2_3	129.48	130.4	1.01	128.65	0.99
90_R2_2	121.18	122.09	1.01	121.89	1.01
90_2_orange part	131.14	132.01	1.01	131.14	1.00
90_2_grey part	142.76	143.63	1.01	142.79	1.00
90_R2	97.94	97.15	0.99	105.94	1.08

Movie 1. Live-cell imaging of fibroblasts on SBS-Sp-pNIPAM hydrogels after 1 round of actuation (1× 5h 90 µm wide grooves).

Movie 2. Live-cell imaging of fibroblasts on SBS-Sp-pNIPAM hydrogels after 2 rounds of actuation (2× 5h 90 µm wide grooves).
